# Effect of CO_2_ Preservation Treatments on the Sensory Quality of Pomegranate Juice

**DOI:** 10.3390/molecules25235598

**Published:** 2020-11-28

**Authors:** Ana Carolina Mosca, Leonardo Menghi, Eugenio Aprea, Maria Mazzucotelli, Jose Benedito, Alessandro Zambon, Sara Spilimbergo, Flavia Gasperi

**Affiliations:** 1 Department of Industrial Engineering, University of Padua, Via Marzolo 9, 35131 Padua, Italy; acmosca@outlook.com (A.C.M.); alessandro.zambon@unipd.it (A.Z.); sara.spilimbergo@unipd.it (S.S.); 2 Department of Food Quality and Nutrition, Research and Innovation Centre, Fondazione Edmund Mach, via E. Mach 1, 38010 San Michele all’Adige, Italy; leonardo.menghi@fmach.it (L.M.); eugenio.aprea@fmach.it (E.A.); mazzucotellimaria@gmail.com (M.M.); 3 Center Agriculture Food Environment, University of Trento/Fondazione Edmund Mach, via E. Mach 1, 38010 San Michele all’Adige, Italy; 4 Department of Technology and Innovation, University of Southern Denmark, Campusvej 55, 5230 Odense, Denmark; 5 Department of Food Technology, Universitat Politècnica de València, Camino de Vera, s/n, 46022 València, Spain; jjbenedi@tal.upv.es

**Keywords:** pomegranate, supercritical carbon dioxide, pasteurization, projective mapping, check-all-that-apply, volatile profile

## Abstract

Due to the interest in identifying cost-effective techniques that can guarantee the microbiological, nutritional, and sensorial aspects of food products, this study investigates the effect of CO_2_ preservation treatment on the sensory quality of pomegranate juice at t_0_ and after a conservation period of four weeks at 4 °C (t_28_). The same initial batch of freshly squeezed non-treated (NT) juice was subjected to non-thermal preservation treatments with supercritical carbon dioxide (CO_2_), and with a combination of supercritical carbon dioxide and ultrasound (CO_2_-US). As control samples, two other juices were produced from the same NT batch: A juice stabilized with high pressure treatment (HPP) and a juice pasteurized at high temperature (HT), which represent an already established non-thermal preservation technique and the conventional thermal treatment. Projective mapping and check-all-that-apply methodologies were performed to determine the sensory qualitative differences between the juices. The volatile profile of the juices was characterized by gas chromatography-mass spectrometry. The results showed that juices treated with supercritical CO_2_ could be differentiated from NT, mainly by the perceived odor and volatile compound concentration, with a depletion of alcohols, esters, ketones, and terpenes and an increase in aldehydes. For example, in relation to the NT juice, limonene decreased by 95% and 90%, 1-hexanol decreased by 9% and 17%, and camphene decreased by 94% and 85% in the CO_2_ and CO_2_-US treated juices, respectively. Regarding perceived flavor, the CO_2_-treated juice was not clearly differentiated from NT. Changes in the volatile profile induced by storage at 4 °C led to perceivable differences in the odor quality of all juices, especially the juice treated with CO_2_-US, which underwent a significant depletion of all major volatile compounds during storage. The results suggest that the supercritical CO_2_ process conditions need to be optimized to minimize impacts on sensory quality and the volatile profile.

## 1. Introduction

Interest in the pomegranate (*Punica granatum L.*) fruit and its products has increased markedly in recent years due to the potential health benefits associated with this fruit. Studies have reported a high level of antioxidant activity in pomegranates [1,2,3], alongside other health benefits such as anti-atherogenic, anti-atherosclerotic, antihypertensive, anti-inflammatory, anti-carcinogenic and anti-angiogenic effects [4,5,6].

Different odors, flavors, and mouthfeel sensations characterize the perception of pomegranate fruit and its products [7]. Koppel and Chambers IV [8] developed a lexicon to describe pomegranate juices in a study that evaluated 33 different products. The list of thirty-four attributes identified includes sour, sweet, musty/earthy, fruity aromatics, astringent mouthfeel, and others. Sugars, mainly fructose and glucose, and acids, mainly citric and malic, are the key contributors to pomegranate sweetness and sourness [9,10,11]. The characteristic astringency mouthfeel perceived during the consumption of pomegranate fruits and products is related to the presence of hydrolyzed tannins, mainly punicalagin [7]. Alcohols, aldehydes, ketones, and terpenes are the main compounds present in the volatile profile of pomegranates [12,13,14,15] and are potentially responsible for its flavor.

There is interest in preserving the nutrient composition and sensorial characteristics of pomegranates during the manufacturing of commercial products, especially juices which are the main processed product of pomegranate fruits. Thermal processing is commonly applied for the preservation of juices due to its cost-effectiveness, easy implementation, and the extensive knowledge available [16]. High temperatures guarantee the inactivation of microorganisms and endogenous enzymes, which allows for an extended shelf-life of commercial products. However, heat treatments can have a negative influence on quality parameters, including the nutritional and functional properties of juices such the color [17,18], vitamins and phytochemicals, and antioxidant activity [17,18,19,20,21]. Different technological approaches have been tested to preserve the overall quality of pomegranate juices, such as ohmic heating [22], high hydrostatic pressure (HPP) [23,24], ultrasound processing [25,26], pulsed electric field processing [27,28], and UV-C irradiation [29]. All these technologies have the potential to ensure microbial safety and preserve the nutritional and sensorial quality, but their operational conditions must be adjusted according to the raw material to yield optimal results [30].

An alternative non-thermal preservation treatment is the processing with supercritical carbon dioxide (CO_2_). At high pressure, CO_2_ exploits bactericidal properties and inactivates microorganisms, mainly due to the modification of the cell’s membrane and a rapid intracellular pH drop [31]. This technology has been shown to guarantee microbial inactivation in different beverages, such as peach and kiwi juices [32], apple juice [33], orange juice [34], and coconut water [35,36], while yielding few changes in the physico-chemical and sensorial properties in relation to untreated products [32,33,34,35,36,37,38]. In terms of the volatile profile, a depletion of volatile compounds has been observed in CO_2_-treated apple juice [33] and coconut water [36,37], which was attributed to a stripping effect during the depressurization at the end of the process. Recently, Bertolini et al. [39] demonstrated that supercritical CO_2_ could be an effective technology to stabilize pomegranate juice, while maintaining its nutritional properties.

This study aims to evaluate the sensorial quality and volatile profile of pomegranate juice treated with CO_2_ alone and in combination with ultrasound (CO_2_-US) at the beginning (t_0_) and end (t_28_) of a storage test at 4 °C. Ultrasound has been successfully coupled to the supercritical CO_2_ process to enhance microbial inactivation and reduce the processing time [40,41,42,43,44]. This synergistic effect can be explained by an accelerated mass transfer of CO_2_ due to the ultrasound [45,46]. To the best of our knowledge this is the first study which applies CO_2_ combined with ultrasound for the preservation treatment of pomegranate juice. As an additional control, we evaluated pomegranate juices treated with high pressure processing (HPP) and pasteurized at high temperature (HT). HPP and HT were chosen because they are, respectively, an already consolidated non-thermal preservation technique and the conventional pasteurization treatment. We hypothesize that the sensorial quality, physico-chemical properties, and odor profile of pomegranate juices treated with supercritical CO_2_ will be less affected due to the use of low temperatures. A modified projective mapping/napping [47,48] protocol in combination with check-all-that-apply (CATA) [49] was used to assess the sensory qualitative differences between juices treated by thermal and non-thermal preservation techniques.

## 2. Materials and Methods 

### 2.1. Sample Preparation

The non-treated juice (NT) was obtained from pomegranates (*Punica granatum*, Wonderful cultivar) using an industrial screw extractor. The fruits were harvested in Spain in September 2018 at the right degree of ripeness required by the industrial squeezing process. Ascorbic acid (0.1% *w*/*w*) was added before homogenization. One batch of NT juice was prepared and subsequently divided into 250 mL bottles, which were kept frozen at −20 °C until further processing. The same initial batch of NT juice was then subjected to thermal and non-thermal treatments: Preservation with supercritical CO_2_ (CO_2_), preservation with a combination of supercritical CO_2_ and ultrasound (CO_2_-US), preservation with high pressure processing (HPP), and pasteurization at high temperatures (HT). Before each treatment, the samples were thawed at 4 °C overnight. Immediately after being treated, all juices were frozen at −20 °C. The freezing steps were necessary as the production of the NT juice, the preservation treatments, and the sensory test were conducted in different locations. We are aware that freezing can induce changes in the juices, but we assumed that these changes would be similar in all juices. The conditions of each treatment are described below.

#### 2.1.1. Supercritical-Carbon Dioxide (CO_2_)

The supercritical CO_2_ treatment was carried out in a continuous laboratory-scale plant at the Polytechnic University of Valencia, Spain. The preservation treatment was done in a reactor consisting of a tank (internal volume of 500 mL) and a holding tube (52 mL). The mixture of juice and supercritical CO_2_ was introduced into the reactor, passed through the holding tube, and finally depressurized down to 5 MPa before entering the separation vessel. Following this, the CO_2_ in the gas phase was separated at the head of the vessel and recirculated to the chiller, while the treated juice was taken in sterile conditions through a valve from the bottom of the vessel. Pressure (12.7 ± 0.5 MPa) and temperature (45 ± 1 °C) were selected from a previous work [39], while residence time (15 ± 1 min) was chosen because no further microbial inactivation was achieved with a longer residence time. To ensure the stationary state of the system, the first 250 mL of juice extracted from the separator were discarded, and only the juice extracted subsequently was used for the analyses. After lamination through the valve, the juice was collected in 250 mL bottles and immediately frozen. To ensure a sterile juice collection, a Bunsen flame was placed close to the outlet of the plant where the juice was bottled.

#### 2.1.2. Supercritical CO_2_ Combined with Ultrasound (CO_2_-US)

The supercritical CO_2_ combined with ultrasound treatment was carried out in the same continuous laboratory-scale plant at the Polytechnic University of Valencia, Spain. The ultrasound system was composed of three parts: a generator that converted the electrical signal into the required power (30 ± 5 W) and frequency, a piezoelectric transducer consisting of two ceramic rings (external diameter of 35 mm, internal diameter of 12.5 mm, thickness of 5 mm) that converted the electrical signal into mechanical vibrations, and a stainless steel sonotrode inserted inside the reactor and in contact with the juice, which transmitted the acoustic energy directly to the product. The conditions of pressure, temperature, and residence time were the same as described above. More details about the CO_2_-US system can be found in a previous work [44].

#### 2.1.3. High Pressure Process (HPP)

HPP was carried out in an industrial plant (Hiperbaric 420, Burgos, Spain). Bottles of 250 mL volume filled with 220 mL of juice were placed in a cylindrical vessel at an initial temperature of 10 °C, and pressurized at 600 MPa for 3 min. Pressurization took place at a constant rate of 200 MPa/min for 3 min, while depressurization was instantaneous. Deionized water at 10 °C was used as a transmitting pressure medium.

#### 2.1.4. Heat Treatment (HT)

This procedure was performed using a commercial pasteurizer (Qb8-4, Roboqbo, Bentivoglio, Bologna, Italy). A total of 6 L of pomegranate juice was treated. The juice was heated up to 90 °C and maintained for 1 min. Subsequently, the juice was cooled to 60 °C and placed in 250 mL bottles.

### 2.2. Storage Test at 4 °C

Part of the juice (3 L of juices HPP, HT and NT in bottles of 250 mL volume and 1.5 L of juices CO_2_ and CO_2_-US in bottles of 500 mL volume) was kept at 4 °C for 24 h for thawing before the start of the storage test, in which the bottles were kept under refrigeration in a cooling incubator at 4 °C for 28 days. After being removed from the storage test, the juices were kept at −20 °C until further analysis.

Juices at the beginning (t_0_) and the end (t_28_) of the storage test at 4 °C were then removed from storage at −20 °C and placed at 4 °C for 36 h, before being used in the sensory test, physico-chemical characterization (color, soluble solid content, pH), and volatile compound analysis. Microbiological analysis was performed on juices at t_0_ and t_28_ for the quantification of microbial load for mesophilic bacteria, yeast, and molds using Plate Count Agar medium (PCA, Sacco, Italy) and Rosa Bengal Agar Istisan 96/35 (RBA, Sacco, Italy), respectively [39].

### 2.3. Sensory Test

#### 2.3.1. Sensory Panel 

A total of 11 assessors (4 females and 7 males, age range: 24−58 years) participated in the study. Eight assessors had previous experience with sensory analysis and three were naïve. All participants gave written informed consent prior to the first session.

#### 2.3.2. Procedure

The participants attended a total of seven sessions of approximately 1.5 h each over a period of seven weeks. Juices were compared using projective mapping/napping, which consists of positioning products in a bi-dimensional space based on the similarities and differences between them [47,48]. We chose to perform projective mapping/napping by sensory modality, where samples were evaluated first on odor by smelling and then on flavor by tasting. In this manuscript, odor refers to the sensations perceived by smelling (orthonasal olfaction), and flavor refers to the combination of sensations perceived by tasting (gustatory, trigeminal and retronasal odor sensations). Projective mapping/napping by modality has been shown to yield results more closely related to the conventional profiling method in comparison to global napping [50,51]. Additionally, partial napping was suggested to be more suitable for the evaluation of large sample sizes [52]. The original projective mapping/napping protocol was modified in our study by the inclusion of training on the methodology and familiarization with the products, which included the generation of a list of attributes to guide the assessor in the description of the samples. Liu et al. [53] reported that these modifications improved the outcome in comparison to the original protocol. A combination with check-all-that-apply (CATA) [49] was chosen for product characterization, because it is an easy and rapid profiling technique that can be reliably performed by naïve consumers and semi-trained assessors, producing qualitative maps close to the ones obtained by descriptive analysis [54,55].

Five training sessions were performed in order to familiarize the assessors with the samples and the sensory evaluation procedures (projective mapping/napping protocol and CATA questionnaire). Additionally, a list of descriptors and definitions was developed during the training sessions (Table 1).

The evaluation of samples was performed in duplicate in two separate sessions. In each session, two sample sets were presented to assessors: the first set consisted of six juices at the beginning of the storage test (t_0_), and the second one of 11 samples with the juices at the beginning (t_0_) and end (t_28_) of the storage test. In both sets, a replication of juice NT was included as a blind reference to monitor the overall panel performance within and between sessions. Juices were presented at T = 15 ± 1 °C in disposable cups coded with three-digit random numbers. Sample presentation order was balanced over assessor and session.

In the first set of six samples (NT, HT, HPP, CO_2_, CO_2_-US plus a replication of NT), assessors were asked first to smell the juices and to position them on a map displayed in a computer screen on the basis of the odor similarities and differences between products. Following this, assessors performed a CATA test by selecting the attributes that were more appropriate to describe each sample (Table 1). After a 3 min break, assessors were asked to rinse the mouth with water and to eat a piece of bread. The same set of six samples was then tasted and positioned on a new map based on flavor attributes, followed by a CATA test. Between samples, panelists had to rinse the mouth with water and eat a piece of unsalted bread.

Once the evaluation of the first six samples set was completed, assessors received the second set of 11 samples (NT, HT, HPP, CO_2_, CO_2_-US at both beginning (t_0_) and end (t_28_) of the storage test, plus a replication of NT_t0_). In this case, the mapping was based only on odor attributes (according to internal sensory laboratory procedures, products subjected to 28 days of storage are not tasted by panelists if not tested for pathogens). Assessors followed the same procedure as described for projective mapping and CATA.

The sessions were carried out in sensory booths at 20 °C under normal (warm or cold) lighting conditions. FIZZ v2.50 (Biosystemès, Couternon, France) was used for data acquisition.

### 2.4. Analysis of Volatile Compounds

Headspace solid-phase microextraction (HS-SPME) and gas chromatography-mass spectrometry (GC-MS) were used for the extraction, separation, and identification of volatile compounds. Analyses were performed in a Clarus 500 GC unit (PerkinElmer AutoSystem XL, Waltham, MA, USA) coupled with a mass spectrometer (TurboMass Gold; PerkinElmer, Waltham, MA, USA) and equipped with a PAL triaxis autosampler (CTC Analytics, Zwingen, Switzerland).

A volume of 3 mL of juice was transferred into a 20 mL vial. Vials were kept at −80 °C until the day of analysis, when they were thawed before the addition of 50 μL of internal standard (2-octanol, 10 mg/L) and a magnetic stir bar. A DVB/Car/PDMS 2 cm fiber (50/30 μm thickness; Supelco, Bellefonte, PA, USA) was used. Before sampling, the vials were equilibrated at 30 °C for 10 min under constant stirring. The triphasic fiber was then exposed for 45 min at 40 °C to the vial headspace under constant stirring.

The compounds were thermally desorbed from the fiber coating into the GC injector port held at 250 °C in splitless mode. An HP-Innowax fused-silica capillary column (30 m, 0.32 mm ID, 0.5 μm film thickness, J&W Scientific Agilent Technologies, Santa Clara, California, USA) was used to perform the analyte separation. Analyses were performed using helium as the carrier gas at a flow rate of 1.5 mL/min. The oven temperature was programmed as follows: 40 °C (3 min) // 4 °C.min^−1^// 220 °C (1 min) // 10 °C.min^−1^// 250 °C (1 min). The transfer line temperature was 220 °C. The mass spectrometer was operated in electron ionization mode (70 eV), with a scan range from m/z 35 to 300.

The peak areas were either calculated from the total ion current (TIC) or estimated from the integrations performed on selected ions. Relative quantification of the odor compounds was achieved using the internal standard method (2-octanol). Peak identification was based on mass spectral interpretation and on the standard library NIST-2014/Wiley.

Each sample (NT, HT, HPP, CO_2_, CO_2_-US at the beginning (t_0_) and end (t_28_) of the storage test) was analyzed in triplicate (different bottles of the same production batch).

### 2.5. Chemical-Physical Characterization of Juices

Color analysis of the samples was performed using a portable colorimeter (Minolta CM-3500d, Tokyo, Japan). The instrument registered the light transmittance of the juices and expressed the results in the CIELAB color system. Internal calibration was performed with an opaque material provided by the manufacturer, and with distilled water. Pomegranate juice samples were poured in quartz cells with 1 mm of optical path. Measurements were carried out at room temperature. Samples were analyzed in triplicate.

Total soluble solids were measured using a digital refractometer DBR 95 (Giorgio Bormac s.r.l, Carpi, Modena, Italy) and expressed as Brix. Measurements were performed in triplicate. The pH of the samples was measured with a digital pH meter (Inolab pH level 1, WTW GmbH, Weilheim, Germany). A single measurement of pH was performed for each sample. These measurements were performed for the five juices (NT, HT, HPP, CO_2_, CO_2_-US) at two points of storage (0 and 28 days).

### 2.6. Data Analysis

#### 2.6.1. Sensory Data

Projective mapping/napping and CATA data were treated separately. Firstly, projective mapping/napping data from the two evaluation sessions were organized in two n x m matrices, where n refers to the samples (n = 6 for the first set of samples; n = 11 for the second set of samples) and m refers to the spatial configurations of each panelist within the two sessions (m = 22). Next, two different multiple factor analysis (MFA) were performed, using data from both sessions, to match the sensory product configurations from the two evaluation sessions. Secondly, attributes from the CATA questionnaire were checked for panel repeatability among the two evaluation sessions through a Gwet AC-1 test [56], as suggested by Meyners and colleagues [57], and then the ones showing to be repeatable were selected for further investigations. Later, the sum of frequencies across assessors and sessions of selected attributes were used to build a contingency table, which was then tested for independence between the rows and columns through a Chi-square test. Finally, a correspondence analysis (CA) was performed to visualize how the products were relatively positioned in a multidimensional space. Sensory data analysis was carried out in R version 2.12.1 (R Development Core Team, 2010), applying functions from the package FactoMineR [58] and SensoMineR [59], and using the script provided by Meyners and colleagues [57] for the Gwet-AC1 test.

#### 2.6.2. Instrumental Data

For the results of the volatile compound analysis and physico-chemical characterization, one-way ANOVA was used to check for the effects of preservation treatments and storage time on volatile compound content, soluble solid content, and color (XLSTAT; Addinsoft, Paris France). Tukey HSD was used as a post-hoc test for differences between mean values. All tests were carried out at a significance level of α = 0.05. Principal component analysis (PCA) of volatile compounds data was computed by the software The Unscrambler 8.5 (CAMO PROCESS AS, Oslo, Norway) after Log-transformation and unit variance scaling of the variables.

## 3. Results and Discussion

### 3.1. Changes in Pomegranate Juice Induced by Preservation Treatment

#### 3.1.1. Sensory Quality 

**Figure 1:**
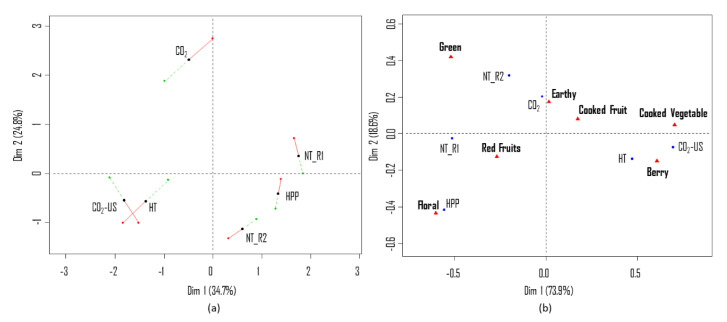
(**a**) MFA individual factor map (first two dimensions) based on odor perception (PM/napping) with superimposed partial points from the two evaluation sessions (S1: red dot and S2: green dot) for each type of preservation treatment considered (NT, HT, HPP, CO_2_, CO_2_-US) at the beginning of the storage test (t_0_). (**b**) CA bi-plot obtained from the CATA questionnaire based on odor perception.

Figure 1a shows the MFA individual factor map of pomegranate juices based on odor perception, according to the PM/napping procedure. The relatively close position of the two blind replications (NT_R1 and NT_R2) reveals a good reliability of the panel over sessions. In the first dimension (34.7%), the variability is mostly explained by differences between the odor of juices that were non-treated (NT) and treated with CO_2_-US, while in the second dimension (24.8%) the variability is mostly explained by odor differences between NT and CO_2_. The close position of HPP and NT in both components indicates that the assessors considered the odor of these juices similar. The distant placement of juices treated with CO_2_ and CO_2_-US in relation to NT suggest that preservation with supercritical CO_2_ affects the odor profile of pomegranate juices. Furthermore, the distant position of juices treated with CO_2_ and CO_2_-US suggests that these samples have a different odor profile. HT and CO_2_-US were positively correlated in both dimensions, indicating a similarity between the odor of these juices. Additionally, the distant position of these two juices in relation to NT in the first dimension indicates that pasteurization at a high temperature and preservation with the combination of CO_2_-US also affect the odor of pomegranate juices. Figure 1a also shows how the same juices were positioned in the two sessions: the overall relationships among samples in the bi-dimensional space were the same for both replicates. The distance between the partial points representing evaluation session 1 (red dot) and evaluation session 2 (green dot) were smaller for samples NT_R1, NT_R2 and HPP than for other samples.

Results from the Gwet-AC1 test revealed seven attributes (berry, cooked fruit, cooked vegetable, green, floral, earthy, red fruits) out of 10 to be repeatable (data not shown). The correspondence analysis bi-plot (Figure 1b) confirms the information previously collected using the projective mapping technique (Figure 1a), with a positive correlation in the first component between juices NT and HPP and between HT and CO_2_-US. The close position of NT_R1 and NT_R2 in the first component, which explains 73.9% of the variability in the data, confirms the reliability of the panel. The juices treated with CO_2_-US and HT were described more frequently as “cooked vegetable” and “berry”. The NT and HPP juices, on the other hand, were described as “floral”, “green” and “red fruits”. The juice treated with CO_2_ was described as “earthy” and “cooked fruit”. These results confirm that panelists perceived differences in the odor of juices treated with CO_2_ and CO_2_-US.

**Figure 2:**
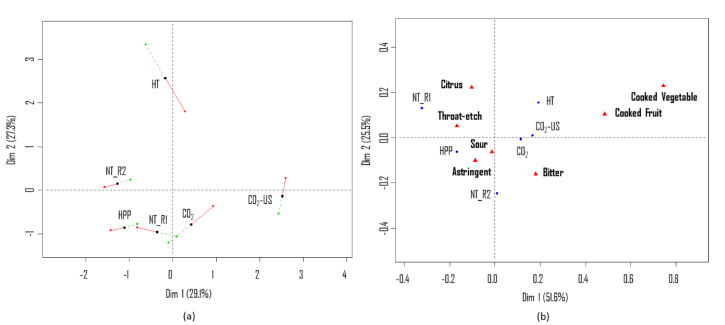
(**a**) Multiple factor analysis (MFA) individual factor map (first two dimensions) based on flavor perception (PM/napping) with superimposed partial points from the two evaluation sessions (S1: red dot and S2: green dot) for each type of pasteurization considered (NT, HT, HPP, CO_2_, CO_2_-US) at the beginning of the storage test (t0). (**b**) CA bi-plot obtained from the CATA questionnaire based on flavor perception.

Figure 2a shows the positioning of juices based on flavor perception. The close position of the blind replications (NT_R1 and NT_R2) confirms the good reliability of the panel. Comparing the two evaluation sessions, a smaller difference was again observed in the placement of NT and HPP. In the first dimension (29.1%), most of the variability is explained by differences between NT and CO_2_-US, while in the second dimension (27.3%), most of the variability is explained by differences between NT and HT. In terms of flavor, HPP was again placed closely to NT in both dimensions, indicating a similarity in flavor between these two juices. HT was distant from NT in the second dimension but was positively correlated with NT in the first dimension, which indicates that this juice could not be clearly differentiated from NT. Regarding juice treated with CO_2_, its difference in relation to NT was larger in the first dimension in session 1. However, in session 2, CO_2_ was positioned close to NT_R1 and HPP in both dimensions, suggesting that the assessors could not differentiate the flavor of these samples.

Results from the Gwet-AC1 test revealed seven attributes (astringent, bitter, citrus, cooked fruit, cooked vegetable, sour, throat-itch) out of 10 to be repeatable (data not shown). In the correspondence analysis bi-plot (Figure 2b), the first component of the model (51.6%) separates the samples based on the attributes “cooked vegetable” and “throat-itch”. The second component (25.5%) discriminates the samples according to the attributes “citrus” and “bitter”. The panel reliability is confirmed by the short distance of the blind references in the first component. The juices treated with CO_2_, CO_2_-US, and HT positively correlated in the first component of the model, and were mainly described as “bitter”. NT and HPP were described as “citrus”, “throat-itch”, “sour” and “astringent”.

Using the projective mapping technique, which is a holistic approach for qualitative differentiation of samples, we observed that the use of supercritical CO_2_ as a preservation technique seems to cause changes in the odor of pomegranate juices, as both juices treated with CO_2_ could be differentiated from the non-treated juice. In terms of flavor however, the differentiation between juices treated with CO_2_, NT, and HPP was not clear. In agreement with Liu et al. [53], our results suggest that projective mapping is a useful and reliable technique that may be used to highlight differences in products with similar sensory characteristics.

Previous studies have reported that beverages treated with supercritical CO_2_ could not be clearly differentiated from non-treated products using discrimination [33,37] and sensory profiling techniques [40]. Damar et al. [35] reported that coconut water treated with CO_2_ could be differentiated from untreated samples in terms of taste and odor. However, these samples did not differ in terms of overall liking and off-flavor. The impact of CO_2_ preservation treatment on the sensory quality of pomegranate juices seems to be more prominent than that observed for apple juice [33] and coconut water [35,37,40]. This indicates that the optimal conditions of CO_2_ treatment that preserve the sensory quality, while ensuring microbiological safety, might depend on the type of product and should be adjusted according to the beverage matrix to minimize changes in the odor and flavor profile, and consequently to product acceptability.

Furthermore, our results suggest that when combined with ultrasound, supercritical CO_2_ has a more prominent impact on the odor quality of pomegranate juices, as the CO_2_-US-treated juice was clearly differentiated from CO_2_-treated juice. The observed differences can be explained by the fact that the same temperature was used for both treatments without considering the synergistic effect of the combination of ultrasound and supercritical CO_2_. As shown by Gao et al. [45] and Riera et al. [46], ultrasound enhances the mass transfer in supercritical CO_2_ extraction, which can induce more prominent changes in the sensorial properties of the juices. This also highlights the need for optimizing the process conditions to guarantee microbiological safety, while preserving the sensorial quality of products treated with CO_2_-US technology.

#### 3.1.2. Physico-Chemical Parameters 

The pH values did not vary due to the preservation treatment (Table 2). Only a slight decrease in the soluble solid content was observed for the CO_2_-US treatment. This might be caused by a limitation of the juice mixing in the separator, where static conditions were maintained and a slight separation could take place before sample collection. These results are in agreement with previous studies that did not observe differences in total soluble solids and pH due to preservation treatments [19,32,33,34,36,37,42,60].

Few changes in color were observed between the juices. L* (lightness) and b* values (blue/yellow) did not vary among the preservation treatments. For a* values (red/green), samples HT and CO_2_-US had a slightly lower redness than NT. Maskan [61] and reported changes in the color parameters of pomegranate juices concentrated using different heating methods, with an increase in darkness (range of L* decrease: 43.4−55.3%), a decrease in redness (range of a* decrease: 19.4−27.2%), and a decrease in yellowness (range of b* decrease: 10.5−21.9%). Similarly, a substantial reduction in all color parameters was observed by Turfan et al. [19] in pasteurized pomegranate juices. The authors attributed these color changes to the degradation of anthocyanin, which is the pigment responsible for the color of pomegranates [19].

Previous studies have suggested that CO_2_ treatment affects the color of different juices by increasing yellowness and lightness and decreasing redness [34,62]. Regarding the combined technology CO_2_-US, studies have shown no effect on the color of dry cured ham [60] and fresh cut carrots [41], while for cooked ham, lightness was higher, redness was lower, and b* did not vary for samples treated with CO_2_-US in comparison to untreated samples [42]. The decrease in redness observed in the current study can be considered negligible, as it would be barely perceptible by the human eye [17,63].

#### 3.1.3. Volatile Compounds

The relative concentrations of the 59 volatile compounds identified in pomegranate juices by SPME/GC-MS are listed in Table 3. Using a consensus approach on different studies that investigated the volatile profile of the pomegranate Wonderful cultivar and several Spanish cultivars (principally Mollar de Elche) [12,13,14,64,65,66], Mayuoni-Kirshinbaum and Pora [7] described the following compounds as the main volatile compounds in pomegranate: Alcohols: hexanol and (Z)-3-hexenol; Aldehydes: hexanal, nonanal and octanal; Ketones: 6-methyl-5- heptene-2-one; and terpenes: β-pinene, limonene, α-terpineol and β-caryophyllene. All these compounds were also identified in the current study, with the exception of sesquiterpene β-caryophyllene. Mayuoni-Kirshinbaum et al. [14] identified the compounds ethyl 2-methylbutanoate, hexanal, β-pinene, (Z)-3-hexenal, β-myrcene, limonene, (Z)-2-heptenal, (Z)-3-Hexenol, 2-ethylhexanol, β-caryophyllene, 2(5H)-furanone, and β-sesquiphellandrene as the odor-active compounds in the Wonderful cultivar of pomegranate fruit using the GC-O technique. According to the authors, these compounds were responsible for the mixture of “green”, “earthy”, “woody”, “fruity”, “floral”, “sweet” and “musty” notes.

**Figure 3:**
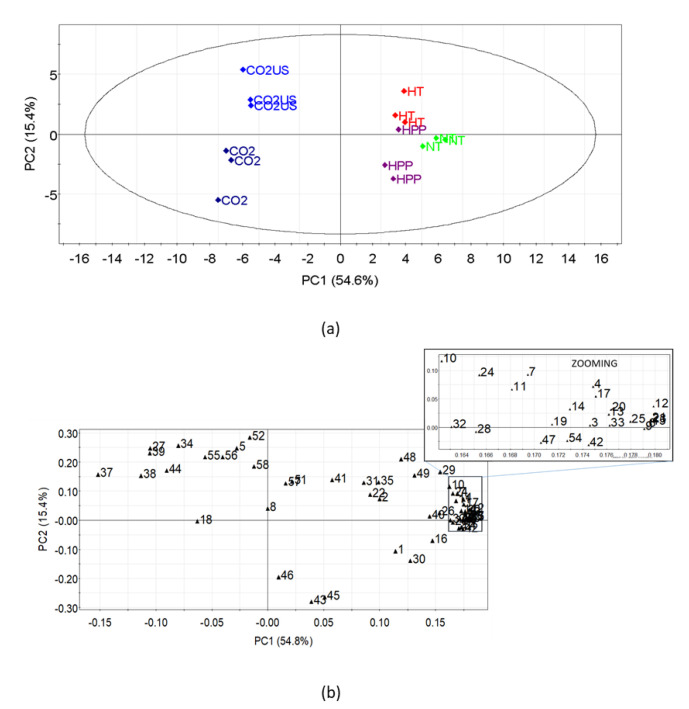
Principal component analysis (PCA) of SPME/GC-MS data for juices at t_0_. (**a**) Score plot of the first two components on standardized results. (**b**) Loading plot of the first two components on standardized results; numbers correspond to the volatile compounds reported in Table 3.

Overall, the concentrations of alcohols, esters, ketones, and terpenes were significantly lower in the CO_2_-treated juices in comparison to the NT juice, while the concentration of aldehydes such as hexanal, (E)-2-hexenal, octanal, nonanal and decanal was higher, especially in the CO_2_-US-treated juice. The volatile compounds present in the highest amounts in the NT juice were limonene, 1-hexanol, and camphene. The first two compounds have been classified as the main volatiles of pomegranates [7], while the latter has not been previously reported in pomegranates. In relation to the NT juice, limonene decreased by 95 and 90%, 1-hexanol decreased by 9 and 17%, and camphene decreased by 94 and 85% in juices treated with CO_2_ and CO_2_-US, respectively. During the decompression stage, when the physical state of CO_2_ changes from liquid to gas, volatile compounds are carried together with CO_2_ (stripping effect), explaining the observed decrease in the concentration of most volatile compounds [37]. These changes in the volatile composition can be better visualized by principal component analysis (Figure 3). A clear separation between juices treated with CO_2_ from the other three juices is shown in Figure 3a. According to the loading plot (Figure 3b), the CO_2_ treatment seems to remove most of the volatile compounds, except for the aldehydes, in juice treated with CO_2_-US. A further difference is the higher amounts of terpenes and sesquiterpenes in CO_2_-US-treated juices compared to the CO_2_ treated juices (Table 3). These differences in volatile compound composition between juices treated with CO_2_ and CO_2_-US might explain the differences in odor observed in the sensory test, which showed that CO_2_-treated juice was more often described as “earthy” and “cooked fruit”, while CO_2_-US was described as “cooked vegetable” and “berry”. The observed differences can be explained by the synergistic effect of the combination of ultrasound and supercritical CO_2_, with ultrasound enhancing the mass transfer in supercritical CO_2_ extraction [45,46], and the effect of ultrasound in accumulating terpenes. In this regard, it has been previously reported that acoustic cavitation, produced by ultrasound, induces volatile compounds to undergo a more rapid degradation compared to less volatile hydrophilic compounds that tend to accumulate in the liquid [67].

Terpenes were identified as the main odor-active compounds that contribute to the pomegranate volatile profile [14]. Furthermore, the presence of monoterpenes such as α-pinene, β-pinene, β-myrcene, limonene, γ-terpinene, and α-terpineol have been shown to correlate positively with overall consumer liking, while the presence of aldehydes such as hexanal has been negatively correlated with consumer liking in studies that compared different pomegranate cultivars [12,13]. The observed effect of CO_2_ preservation treatment in the depletion of terpenes, together with the increase in the concentration of aldehydes, might compromise the sensory performance of pomegranate juices treated with CO_2_.

Previous studies have also observed a depletion of volatile compounds in beverages treated with CO_2_. Gasperi et al. [33] reported a decrease in volatile compounds, especially esters (acetates) and aldehydes, in apple juice treated with supercritical CO_2_ and N_2_O. The authors concluded that the differences in volatile composition could explain the sensorial differences observed in a triangle test. A reduction in the volatile compounds due to supercritical CO_2_ has also been reported in coconut water [36,37]. However, De Marchi et al. [37] observed that the changes in volatile composition induced by CO_2_ treatment did not reflect sensorial differences in relation to untreated samples.

Regarding the other preservation and pasteurization treatments, the pasteurized juice (HT) presented an overall reduction in the concentration of esters in comparison with the non-treated juice, and an increase in the concentration of some aldehydes, like hexanal (16% increase), octanal (220% increase), and benzaldehyde (82% increase). Some odor-active terpenes were found to be reduced in heat-treated samples, especially β-pinene (62%), β-myrcene (19%), and limonene (22%). Other terpenes, like α-phellandrene, α-terpinene and α-curcumene, were present in a slightly higher concentration in HT. The HPP juice also had an overall lower concentration of esters in comparison to NT and many terpenes, including β-pinene (40%) and β-myrcene (26%). The aldehyde profile of HPP was similar to NT, with the exception of the increase in hexanal, (E)-2-hexenal, and benzaldehyde.

The high concentration of the odor-active compounds of pomegranates, such as ethyl 2-methylbutanoate (fruity, apple), 1-hexanol (resin, flower, green), (Z)-3-hexen-1-ol (moss, fresh), β-pinene (pine, resin, turpentine), β-myrcene (woody, musty), and limonene (lemon, orange) in the non-treated juice, can explain the CATA results wherein NT was frequently described as “green” in odor and “citrus” in flavor. On the other hand, the high concentration of aldehydes like hexanal (grass, tallow, fat), octanal (rancid, soapy) and nonanal (fat, citrus and green) in CO_2_-treated samples can explain the description of the odor of CO_2_ as “earthy” and “cooked fruit” and that of CO_2_-US as “cooked vegetable”.

### 3.2. Changes in Pomegranate Juice Induced by Storage 

The results from microbiological analyses showed that the microbial load in non-treated pomegranate juice was 4.3 (t_0_) and 4.6 (t_28_) Log CFU/mL for mesophilic bacteria and 4.2(t_0_) and 4.6 (t_28_) Log CFU/mL for yeast and molds. The microbial load for mesophilic bacteria and yeasts and molds of juices treated with HPP and HT was under the detection limit (< 2 Log CFU/mL) during the 28 day storage at 4 °C. Juices treated with CO_2_-and CO_2_-US were also under the detection limit (<2 Log CFU/mL) for yeasts and molds during storage, while the mesophilic bacteria count was below 2.4 log CFU/mL. 

#### 3.2.1. Sensory Quality

**Figure 4:**
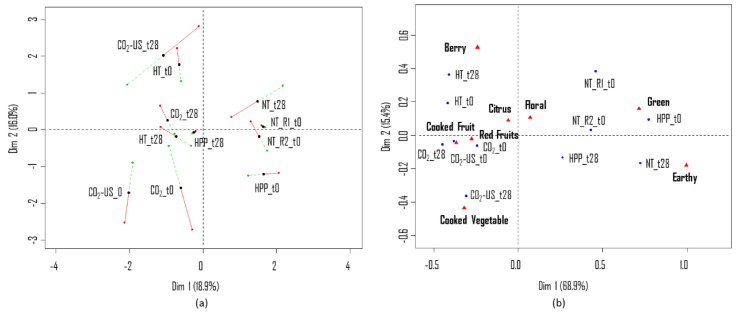
(**a**) MFA individual factor map (first two dimensions) based on odor perception with superimposed partial points from the two evaluation sessions (S1: red dot and S2: green dot) for each type of juice considered (NT, HT, HPP, CO_2_, CO_2_-US) at the beginning (t_0_) and end (t_28_) of storage at 4 °C. (**b**) CA bi-plot obtained from the CATA questionnaire based on odor perception.

Figure 4a shows the configuration of samples at the beginning (t_0_) and at the end (t_28_) of storage, based on odor perception. The close position of the two blind replications (NT_R1 and NT_R2) in both components reveals good inter-session reliability of the panel. Comparing the two evaluation sessions (red and green dots), we observed consistent results over sessions, with a larger variation in the placement of samples for juices CO_2_-US_t_0_, CO_2_-US_t_28_, CO_2__t_0_ and NT_t_28_. In the first dimension, which explains 18.9% of the variability, the separation of samples was based on the preservation technology, with NT and HPP juices being positively correlated and HT, CO_2_, CO_2_-US being oriented oppositely. For the second dimension, which explains 16% of the variability, samples were separated based on storage time, with samples at t_0_ being positioned at the bottom of the map and samples at t_28_ at the top, except for juice HT. The largest difference between samples at t_0_ and t_28_ was observed for CO_2_-US-treated juice (specially in session 1), suggesting that this preservation technique does not guarantee the stability of the volatile profile of the juices overtime. In session 2, juices treated with CO_2_ at t_0_ and t_28_ were positioned closely in terms of both components, which suggests that the volatile profile of this juice did not vary considerably during storage. These results are in agreement with Fabroni et al. [68], who concluded that preservation with supercritical CO_2_ could guarantee the stability of the sensorial quality of blood orange juice for up to 20 days of storage at 4 °C. The results suggested that sensorial deterioration due to spoilage started at around 20−25 days, with a decrease in the attributes freshness, flavor, intensity of taste, and intensity of scent, and a significant increase in off-flavor.

For both HT and HPP juices, the distant position between the samples at t_0_ and t_28_ indicates that the odor of these juices varied during storage. In a study that compared the shelf-life stability of volatile compounds of apple juices treated by Pulsed Electric Field (PEF), HPP, and thermal pasteurization, Kebede et al. [69] observed that immediately after treatment the decrease in the amount of odor-active esters and aldehydes was more prominent in samples submitted to thermal processing in comparison to PEF and HPP technologies. During the shelf-life at 4 °C, however, the concentration of volatiles decreased in a similar fashion in all processed juices. This could explain the sensory results observed in the current study. The close placement of juice HPP_t_0_ and NT_t_0_ in Figure 4a (also HPP and NT in Figure 1a) suggests that the volatile profile of untreated pomegranate juices was preserved by non-thermal preservation treatments, while thermal pasteurization led to perceivable changes in odor (e.g., distant position of HT_t_0_ and NT_t_0_ in Figure 4a and HT and NT in Figure 1a). During the storage test however, the non-thermal treated samples also underwent changes in the volatile composition which could be perceived by the panel. Results from the projective mapping suggests that assessors could not differentiate the odor of the non-treated juices at the two points of storage.

Results from the Gwet-AC1 test revealed eight attributes (“earthy”, “berry”, “cooked fruit”, “cooked vegetable”, “green”, “floral”, “earthy”, “red fruits”) out of 10 to be repeatable (data not shown). The map generated using the data from the CATA questionnaire (Figure 4b) confirms the information previously collected using the projective mapping technique (Figure 4a). The first component of the model (68.9%) separates the samples according to the preservation technology, whereas the second component (15.4%) separates the juices according to storage time, with the exception of CO_2_-treated juice. This juice was more frequently described as “cooked vegetable” and “red fruits”, regardless of storage time. The NT and HPP samples, on the other hand, were described more frequently as “green” and “earthy”. The effect of the storage time on the sensory profiles of juices can be evidenced by the increase in the frequency of the attribute “cooked vegetable” for CO_2_-US-treated juice, “berry” for HT, and “earthy” for NT. 

#### 3.2.2. Effect on the Chemical Physical Parameters

No differences in pH and Brix were observed between juices t_0_ and t_28_ for any of the preservation treatments (Table 4). These results are in agreement with Mayuoni-Kirshinbaum and Porat [66], who reported that the total soluble solids and acidity of pomegranate arils did not vary for up to 8 and 16 weeks of storage at 7 °C, respectively. All color parameters of the heat-treated juice were affected by storage, with an increase in lightness and a decrease in redness and yellowness. For the NT and CO_2_-treated juices, redness decreased and yellowness increased after 28 days at 4 °C. For the HPP juice, only an increase in lightness was observed. Pérez-Vicente et al. [63] reported an overall increase in color lightness, decrease in redness, and slight increase in yellowness of pasteurized pomegranate juices during storage at 24/18 °C. The decrease in a* value was highly correlated with a decrease in anthocyanin concentration. A loss of red color of pomegranate juices (measured as absorbance values at 520 nm) and an increase in the browning index during storage were observed by Vegara et al. (2013) [70]. These changes were less prominent when juices were stored at 5 °C in comparison to at 25 °C. Additionally, changes in color at under 5 °C storage for 120 days were lower for both cloudy and clarified pomegranate juices that underwent low-temperature pasteurization (65 °C for 30 s) in comparison to high-temperature pasteurization (90 °C for 5 s), with a loss of 55 and 75% (cloudy) and 40 and 53 % (clarified) for low- and high-temperature pasteurization, respectively.

#### 3.2.3. Effect on Volatile Compounds

The variations in concentration of the 59 volatile compounds identified in pomegranate juices at the two points of storage (t_0_ and t_28_) are listed in Table 5.

**Figure 5:**
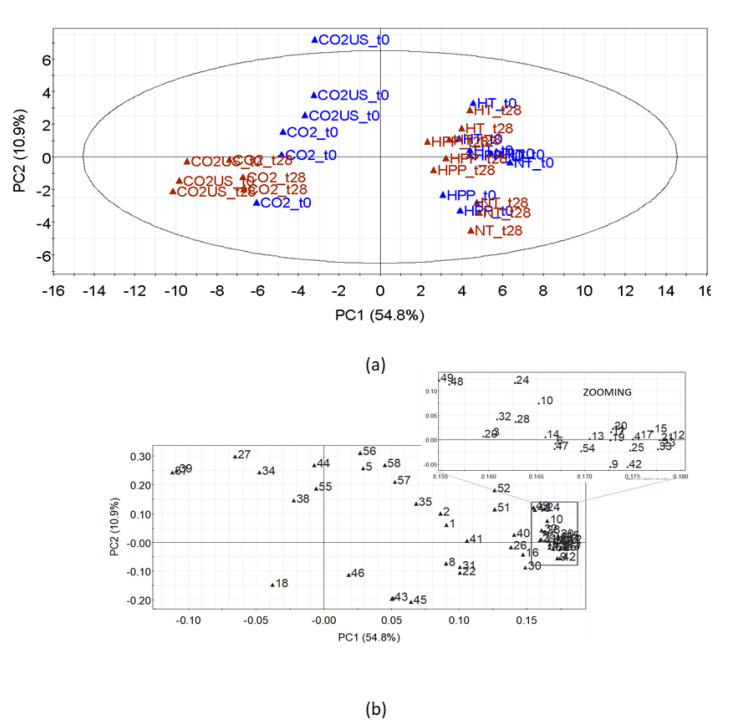
PCA of SPME/GC-MS data for juices at t_0_ and t_28_. (**a**) Score plot of the first two components on standardized results. (**b**) Loading plot of the first two components on standardized results; numbers correspond to volatile compounds reported in Table 3.

The effect of storage on the concentration of the main volatile compounds of pomegranate varied according to the preservation technology. The concentration of 1-hexanol (resin, flower, green) considerably decreased with storage time for HPP and CO_2_-US-treated juices, and increased for NT. The concentration of (Z)-3-hexen-1-ol (moss, fresh) decreased for NT and CO_2_-US. The concentration of hexanal (grass, tallow, fat) decreased for all technologies, with the exception of NT which showed an increase in the concentration of this aldehyde. Octanal (rancid, soapy) decreased for HT and CO_2_-US, while the concentration of nonanal was not significantly affected by storage time in any of the preservation technologies. The ketone 6-methyl 5-hepten-2-one (oil, herbaceous, green) significantly increased in NT and decreased in CO_2_-US. The concentration of terpenes β-pinene (pine, resin, turpentine) and limonene (lemon, orange) decreased for all preservation technologies, while the concentration of α-terpineol (oil, anise, mint) increased for HT and decreased only for CO_2_-US. Additionally, the concentration of the odor-active compound β-myrcene (woody, musty) decreased in the HPP and CO_2_-treated juices. 2-ethylhexanol (floral) increased in HPP and HT and decreased in CO_2_-US. β-sesquiphellandrene (terpene, almond) increased in NT and decreased in CO_2_-treated juices. Ethyl 2-methylbutanoate (fruity, apple) decreased for NT and CO_2_-US, and increased for HPP. The effect of storage on the volatile composition is shown in the PCA plot (Figure 5).

Overall, the changes in the volatile profile of main compounds might explain the differences induced by storage detected by the sensory panel for CO_2_-treated juices. The CO_2_-US-treated juice especially showed a significant depletion of all the main volatiles and odor-active compounds of pomegranates (with exception of nonanal), suggesting that this technology does not guarantee the stability of the volatile profile of the juices overtime. This result is in agreement with the projective mapping, in which the assessors perceived a large difference between juices treated with CO_2_-US at t_0_ and t_28_ (Figure 4a).

Even though NT at t_0_ and t_28_ could not be clearly differentiated in the projective mapping, in the CATA test NT_t_28_ was described as “green” and “earthy”. This might be explained by the significant increase in the concentration of 6-methyl 5-hepten-2-one, hexanal, (E)-2-hexenal, and 1-hexanol, which are responsible for fresh, green grass and herbaceous notes, as well as the increase in the concentration of terpenes such as α-pinene and β-sesquiphellandrene.

Differences in the volatile profile of HPP at t_0_ and t_28_ were perceived by the sensory panel. The decrease in the concentration of volatile compounds, as listed above, together with an increase in 2-ethylhexanol, α-pinene, ethyl 2-methylbutanoate and 4-terpineol, could explain the differences perceived. For the heat pasteurized sample, the increase in the concentration of 2-ethyl-1-hexanol and 4-terpineol at the end of the storage test might explain the description of HT_t_28_ as “berry”. 

In a study that investigated the changes in sensory quality and volatile composition of pomegranate arils after up to 20 weeks of storage at 7 °C, Mayuoni-Kirshinbaum and Porat [66] observed that a decrease in flavor preference was mainly due to the increase in the intensity of the attributes “overripe” and “off-flavour”. Additionally, a decrease in flavor preference was highly correlated with the increase in the accumulation of ethanol and its esterification product ethyln acetate, and the accumulation of sesquiterpene volatiles such as β-caryophyllene, α-curcumene, (E)-α-bergamotene, (E)-β-farnesene, (Z)-β-farnesene, and β-sesquiphellandrene. Of these compounds identified as contributors to negative changes in pomegranates during storage, only ethyl acetate, α-curcumene, and β-sesquiphellandrene were identified in pomegranate juices in the current study. The concentration of ethyl acetate decreased with storage in NT and CO_2_-US, and significantly increased in HPP. α-curcumene significantly decreased over time in the CO_2_-treated juices. The concentration of β-sesquiphellandrene, which is an odor-active compound of pomegranates, increased in NT and decreased in CO_2_-treated juices.

It is worth emphasizing that the results observed in this study are related to the processing conditions and storage test temperature employed and might not be able to be extrapolated to different treatment conditions. The authors acknowledge that comparisons between preservation techniques should be done with caution, especially when only one condition per treatment is compared. Additionally, the authors emphasize that the outcomes reported refer to treatments performed at a laboratory scale, which might not be reproducible at large scales.

## 4. Conclusions

As a closing remark, the preservation treatment with supercritical CO_2_ led to perceivable changes in the odor and volatile profile of pomegranate juices, especially when the supercritical CO_2_ was combined with ultrasound. As supercritical CO_2_ has been shown to be a feasible technique for the preservation of beverages (e.g., apple juice and coconut water) without leading to major changes to the sensorial properties, our results suggest that the optimal process parameters might be product dependent. This means the conditions of the preservation treatment with supercritical CO_2_ should be adjusted according to the product to minimize changes in the sensorial quality.

It is important to highlight that both juices treated with CO_2_ were obtained in a lab-scale plant using process conditions based only on microbial and nutritional stability criteria. Further studies are required to check whether the results obtained at the lab-scale could be reproduced at a large scale. The process represents a new and promising approach with potential for improvement, which could lead to better sensorial characteristics of the product. Moreover, to the best of our knowledge, a CO_2_-US pilot plant still does not exist. Further investment is needed to promote the development of a pilot plant that combines the two technologies, which could facilitate comparison with other preservation technologies at a similar industrial scale. Additionally, storage tests at 6−8 °C are required to estimate the shelf-life of CO_2_-treated juices at commercial storage temperatures.

## Figures and Tables

**Table 1:** Attribute list used for the sensory evaluation of pomegranate juices treated with different preservation techniques.

Attribute	Description
Odor	Olfactory sensations perceived by smelling (orto-nasally)
Citrus	Sensation that recalls the smell of citrus fruits (lemon, orange, grapefruit)
Green	Sensation that recalls the smell of freshly cut grass
Floral	Sensation that recalls the smell of flowers
Unripe fruit	Sensation that recalls the smell of the white film that covers the seed of pomegranate and the smell of unripe fruit
Cooked fruit	Sensation that recalls the smell of cooked fruit
Berry	Sensation that recalls the smell of wild berries (blueberry, blackberry, mulberry, black currant)
Red fruits	Sensation that recalls the smell of red fruits (cherry, raspberry, gooseberry)
Earthy	Sensation that recalls the smell of wet earth
Fresh vegetable	Sensation that recalls the smell of green vegetables and fresh green vegetable stalks
Cooked vegetable	Sensation that recalls the smell of cooked vegetables (green beans, potatoes)
Taste	
Sweet	Basic taste typical of sucrose (e.g., sugar)
Bitter	Basic taste typical of quinine (e.g., coffee)
Sour	Basic taste typical of citric acid (e.g., lemon)
Flavor	Odors perceived through the mouth (retro-nasally)
Citrus	Sensation associated with citrus fruits (lemon, orange, grapefruit)
Unripe fruit	Sensation associated with the white film covering the seed of pomegranate and with unripe fruit
Cooked fruit,	Sensation associated with cooked fruit
Cooked vegetable	Sensation associated with cooked vegetables (beans, potatoes)
Mouthfeel/sensation	
Astringent	Sensation of dry, puckering, roughing mouthfeel
Pungent	Tingling sensation on the tongue not associated with a sensation of heat
Throat-itch	Pricking sensation felt only in the throat and not associated with a sensation of heat

**Table 2:** Soluble solid content (SSC), pH, and color parameters of pomegranate juices (mean values and standard deviation) and ANOVA *p*-values (*p*).

	NT	HPP	HT	CO_2_	CO_2_-US	*p*
SSC (brix)	16.6 (0.1)a	16.6 (0.2)a	16.5 (0.2)ab	16.3 (0.1)ab	16.2 (0.2)b	0.005
pH	3.1	3.1	3.1	3.2	3.2	-
L*	45.4 (1.9)	42.5 (3.2)	45.6 (0.3)	44.9 (2.5)	44.2 (4.1)	0.096 (NS)
a*	63.3 (1.4)a	60.8 (3.4)ab	59.1 (0.2)b	61.2 (1.7)ab	59.3 (3.7)b	0.007
b*	14.3 (3.1)	17.7 (2.4)	16.1 (0.3)	15.7 (1.8)	17.4 (4.0)	0.059 (NS)

a, b Means containing the same letter within a row are not significantly different (*p* < 0.05). NS: not significant.

**Table 3:** Volatile compounds identified in the pomegranate juices at t = 0 of storage: For each compound, listed in order of linear retention index (LRI), we give the mean values related to the five preservation treatments with standard deviations in brackets and the *p*-values (*p*) of one-way ANOVA applied to check for preservation treatment effects.

			Concentration Volatile Compounds (µg/L of 2-Octanol)					
	Compound	LRI	NT	HPP	HT	CO_2_	CO_2_-US	
1	ethyl acetate	898	16 (1)a	6.9 (0.3)d	9.33 (0.04)b	8.4 (0.4)c	5.2 (0.5)e	<0.0001
2	2-pentanone	988	0.38 (0.01)c	1.08 (0.03)b	2.19 (0.09)a	0.30 (0.03)d	0.33 (0.02)d	<0.0001
3	methyl 2-methylbutanoate	1019	3.1 (0.2)a	1.25 (0.06)c	1.91 (0.02)b	0.29 (0.06)d	0.33 (0.02)d	<0.0001
4	α-pinene	1024	16.3 (1.6)a	6.5 (0.7)b	14 (1)a	1.2 (0.1)d	2.6 (0.4)c	<0.0001
5	toluene	1048	0.28 (0.06)b	0.3 (0.1)b	0.21 (0.02)b	0.10 (0.04)c	0.7 (0.2)a	0.0001
6	ethyl 2-methylbutanoate	1060	8.5 (0.5)a	4.5 (0.1)b	4.6 (0.1)b	0.9 (0.1)d	1.4 (0.1)c	<0.0001
7	camphene	1066	93.5 (9.8)a	26 (2)c	73 (4)b	5.3 (0.2)e	14 (2)d	<0.0001
8	hexanal	1093	1.9 (0.1)c	2.7 (0.3)a	2.2 (0.1)b	2.02 (0.03)c	2.4 (0.2)ab	0.001
9	β-pinene	1111	25.8 (1.8)a	16 (1)b	10 (1)c	0.32 (0.07)e	1.4 (0.2)d	<0.0001
10	isoamyl acetate	1133	3.6 (0.3)a	1.3 (0.2)b	3.8 (0.4)a	ND	0.9 (0.1)b	<0.0001
11	α-phellandrene	1171	4.2 (0.4)b	2.8 (0.4)c	7.1 (0.7)a	0.42 (0.09)e	0.71 (0.07)d	<0.0001
12	β-myrcene	1174	16.1 (1.2)a	11.9 (0.8)b	13 (1)b	1.1 (0.1)d	2.8 (0.3)c	< 0.0001
13	α-terpinene	1187	2.7 (0.3)b	2.1 (0.1)c	3.7 (0.2)a	0.09 (0.02)e	0.17 (0.03)d	<0.0001
14	methyl hexanoate	1199	2.5 (0.1)a	0.94 (0.09)c	1.70 (0.08)b	0.18 (0.05)e	0.32 (0.02)d	<0.0001
15	limonene	1206	460 (29)a	444 (11)a	359 (30)b	24.5 (0.4)d	46 (5)c	<0.0001
16	1.8-cineole	1213	4.6 (0.3)a	3.3 (0.1)c	3.8 (0.1)b	3.1 (0.2)c	2.6 (0.1)d	<0.0001
17	β-phellandrene	1215	37 (5)a	32 (1)a	30 (4)a	5.6 (0.3)c	12 (1)b	<0.0001
18	(E)-2-hexenal	1228	ND	0.5 (0.1)a	0.29 (0.04)b	0.3 (0.1)b	0.37 (0.08)ab	<0.0001
19	2-pentyl furan	1244	0.86 (0.04)a	0.72 (0.02)b	0.48 (0.06)c	0.06 (0.02)e	0.24 (0.02)d	<0.0001
20	ethyl hexanoate	1245	4.6 (0.3)a	2.22 (0.06)c	2.63 (0.06)b	0.45 (0.08)e	0.92 (0.05)d	<0.0001
21	γ-terpinene	1254	53 (3)a	52 (2)a	45 (4)b	1.4 (0.1)d	3.8 (0.3)c	<0.0001
22	styrene	1269	0.30 (0.07)a	0.23 (0.04)a	0.07 (0.01)b	ND	0.22 (0.04)a	<0.0001
23	p-cymene	1280	17 (1)a	16.0 (0.4)a	12.6 (0.7)b	0.8 (0.1)d	2.0 (0.1)c	<0.0001
24	hexyl acetate	1284	1.50 (0.06)a	0.87 (0.07)b	1.00 (0.06)b	0.21 (0.07)d	0.6 (0.1)c	<0.0001
25	terpinolene	1292	3.9 (0.3)a	4.4 (0.2)a	4.4 (0.3)a	0.15 (0.03)c	0.47 (0.05)b	<0.0001
26	2-octanone	1296	3.8 (0.1)a	3.9 (0.3)a	3.67 (0.03)a	3.2 (0.1)b	3.34 (0.08)b	0.002
27	octanal	1300	0.42 (0.07)c	0.4 (0.2)c	1.28 (0.02)b	0.9 (0.2)b	2.0 (0.2)a	<0.0001
28	(Z)-3-hexen-1-ol acetate	1330	1.38 (0.04)a	0.77 (0.07)b	0.78 (0.03)b	0.36 (0.03)d	0.46 (0.04)c	<0.0001
29	6-methyl 5-hepten-2-one	1350	1.47 (0.05)a	1.13 (0.07)b	1.47 (0.05)a	0.7 (0.1)c	1.1 (0.1)b	<0.0001
30	1-hexanol	1365	86 (6)bc	108 (4)a	91.7 (0.3)b	78 (2)cd	72 (6)d	<0.0001
31	(E)-3-hexen-1-ol	1375	3.3 (0.3)bc	4.05 (0.06)a	4.2 (0.1)a	3.0 (0.2)c	3.7 (0.1)b	<0.0001
32	(Z)-3-hexen-1-ol	1396	1.53 (0.09)a	0.73 (0.06)b	0.74 (0.01)b	0.28 (0.04)d	0.39 (0.05)c	<0.0001
33	2-nonanone	1399	11.9 (0.6)a	11.8 (0.4)a	8.62 (0.02)b	2.5 (0.1)d	4.1 (0.2)c	<0.0001
34	nonanal	1405	3.2 (0.6)bc	2.7 (0.4)c	4.0 (0.3)b	3.4 (0.5)bc	5.7 (0.9)a	0.001
35	furfural	1478	2 (2)	1.1 (0.4)	3.9 (0.2)	0.12 (0.03)	2 (2)	0.073 (NS)
36	tetramethylbenzene 1,2,3,4	1498	ND	ND	ND	1.15 (0.01)a	0.82 (0.07)b	<0.0001
37	2-ethyl-1-hexanol	1502	1.16 (0.06)e	1.6 (0.2)d	3.0 (0.05)c	3.78 (0.05)b	6.1 (0.2)a	<0.0001
38	decanal	1510	1.0 (0.1)bc	0.7 (0.2)c	1.1 (0.2)bc	1.2 (0.1)b	1.6 (0.1)a	0.001
39	benzaldehyde	1539	1.13 (0.06)b	0.5 (0.1)c	2.03 (0.09)a	1.8 (0.4)a	2.9 (0.7)a	0.0001
40	linalool	1559	0.82 (0.08)b	0.95 (0.1)b	1.26 (0.04)a	0.56 (0.07)c	0.44 (0.09)c	<0.0001
41	4-terpineol	1606	0.56 (0.07)b	ND	0.84 (0.02)a	0.3 (0.1)c	0.27 (0.04)c	<0.0001
42	2-octen-1-ol acetate	1639	2.38 (0.3)a	2.4 (0.3)a	1.4 (0.2)b	0.18 (0.06)d	0.40 (0.06)c	<0.0001
43	1-hexadecene	1651	1.0 (0.2)ab	1.5 (0.8)a	0.8 (0.5)ab	1.4 (0.3)ab	0.20 (0.06)b	0.040
44	acetophenone	1662	0.2 (0.1)	0.12 (0.04)	0.3 (0.2)	0.5 (0.3)	0.7 (0.7)	0.4 (NS)
45	unidentified hydrocarbon	1674	1.8 (0.2)	5 (5)	3 (3)	3 (2)	0.2 (0.1)	0.324 (NS)
46	heptadecane	1700	ND	0.6 (0.6)	0.3 (0.3)	0.3 (0.1)	ND	0.195 (NS)
47	α-terpineol	1706	1.8 (0.1)ab	1.67 (0.06)b	2.05 (0.08)a	1.02 (0.07)c	0.83 (0.04)d	<0.0001
48	zingiberene	1730	1.1 (0.1)b	1.1 (0.2)b	1.43 (0.04)a	0.71 (0.08)c	1.0 (0.1)b	0.0001
49	β-bisabolene	1737	0.48 (0.02)b	0.6 (0.1)ab	0.7 (0.1)a	0.29 (0.09)c	0.44 (0.05)bc	0.01
50	naphthalene	1751	0.14 (0.06)c	0.3 (0.1)c	0.3 (0.1)c	2.12 (0.08)a	1.44 (0.04)b	<0.0001
51	β-sesquiphellandrene	1779	0.43 (0.02)	0.5 (0.1)	0.51 (0.04)	0.45 (0.09)	0.48 (0.05)	0.6 (NS)
52	α-curcumene	1784	0.91 (0.07)c	1.0 (0.1)bc	1.26 (0.05)a	1.0 (0.1)bc	1.18 (0.03)ab	0.006
53	3.5-dimethylbenzaldehyde	1822	0.14 (0.04)d	0.31 (0.08)c	0.33 (0.09)c	1.92 (0.09)b	2.7 (0.2)a	<0.0001
54	anethole	1840	0.55 (0.04)a	0.40 (0.03)b	0.28 (0.03)c	0.05 (0.02)e	0.10 (0.02)d	<0.0001
55	hexanoic acid	1876	0.98 (0.05)ab	1.03 (0.07)ab	0.88 (0.04)b	0.9 (0.2)ab	1.4 (0.4)a	0.048
56	Phenol	2018	0.15 (0.03)	0.14 (0.02)	0.16 (0.04)	0.15 (0.06)	0.2 (0.1)	0.653 (NS)
57	p-cresol	2094	0.09 (0.02)	0.10 (0.03)	0.09 (0.03)	0.09 (0.04)	0.09 (0.04)	0.98 (NS)
58	m-cresol	2102	0.53 (0.08)	0.54 (0.05)	0.56 (0.09)	0.5 (0.1)	0.6 (0.2)	0.96 (NS)

Means containing the same letter within a row are not significantly different (*p* < 0.05). ND: not detected. NS: not significant.

**Table 4:** Variation in soluble solid content (SSC), pH, and color parameters between pomegranate juices at t_0_ and t_28_ (Δ*), and ANOVA *p*-values (*p*).

	ΔNT	*p*	ΔHPP	*p*	ΔHT	*p*	ΔCO_2_	*p*	ΔCO_2_-US	*p*
SSC (brix)	0.1	0.519	0.3	0.065	0.03	0.815	−0.1	0.116	0.2	0.057
pH	0	-	0	-	0	-	0	-	0	-
L*	−0.5	0.608	4.4	0.006	4.1	<0.0001	−0.8	0.495	0.8	0.669
a*	−4.6	0.0001	−1.2	0.399	−5.5	<0.0001	−5.4	<0.0001	−4.1	0.033
b*	2.9	0.051	−2.1	0.056	−1.2	<0.0001	3.8	0.001	4.4	0.031

Δ* indicates variation between juices at t_0_ and t_28_ within the same preservation treatment. Delta was calculated as the difference between the average values (for the three replicates) at t_0_ and t_28_.

**Table 5:** Variation in concentration of volatile compounds between pomegranate juices at t_0_ and t_28_ (Δ*) and ANOVA *p*-values (*p*).

	ΔNT	*p*	ΔHPP	*p*	ΔHT	*p*	ΔCO_2_	*p*	ΔCO_2_-US	*p*
ethyl acetate	−11.10	<0.0001	3.61	0.001	0.03	0.871	−0.44	0.436	−1.21	0.015
2-pentanone	0.01	0.422	−0.74	0.001	−0.06	0.442	−0.02	0.506	0.09	0.025
methyl 2-methylbutanoate	−1.88	0.0001	1.68	0.0001	0.10	0.182	0.003	0.945	−0.14	0.002
α-pinene	3.22	0.029	5.61	0.001	−1.54	0.122	−0.80	0.0001	−2.22	0.0001
toluene	−0.06	0.243	−0.10	0.239	0.11	0.015	−0.01	0.819	−0.47	0.007
ethyl 2-methylbutanoate	−4.58	<0.0001	3.27	0.001	0.35	0.009	0.16	0.295	−0.63	0.011
camphene	22.21	0.020	35.20	0.0001	−5.71	0.134	−2.50	<0.0001	−11.35	0.001
hexanal	1.46	0.003	−1.01	0.014	−0.32	0.029	−0.28	0.044	−1.01	0.001
β-pinene	−8.65	0.001	−6.25	0.002	−4.66	0.001	−0.32	0.001	−1.31	0.0001
isoamyl acetate	−1.65	0.002	2.07	<0.0001	−0.42	0.182	DL	-	−0.85	0.0001
α-phellandrene	0.80	0.028	0.27	0.326	−1.38	0.024	−0.21	0.013	−0.71	<0.0001
β-myrcene	−0.25	0.783	−2.30	0.013	−0.35	0.656	−0.87	0.0001	−2.80	<0.0001
α-terpinene	−0.46	0.036	−0.17	0.074	0.15	0.384	−0.05	0.020	−0.17	0.001
methyl hexanoate	−1.04	0.0001	1.39	<0.0001	−0.03	0.682	−0.03	0.449	−0.21	0.003
limonene	−133.48	0.001	−120.22	0.001	−27.38	0.199	−19.96	<0.0001	−43.35	0.0001
1.8-cineole	0.54	0.043	0.42	0.021	−0.01	0.961	0.03	0.820	−1.53	<0.0001
β-phellandrene	11.71	0.015	−12.38	0.002	−5.07	0.075	−2.83	0.0001	−10.75	0.0001
(E)-2-hexenal	0.83	<0.0001	−0.33	0.005	0.11	0.221	0.23	0.024	0.11	0.310
2-pentyl furan	−0.30	0.001	−0.27	<0.0001	0.05	0.317	−0.06	0.003	−0.24	<0.0001
ethyl hexanoate	−2.32	0.0001	1.26	0.0001	−0.09	0.252	−0.19	0.019	−0.75	<0.0001
γ-terpinene	−16.68	0.001	−15.61	0.001	−4.62	0.105	−1.26	<0.0001	−3.71	<0.0001
styrene	0.41	0.161	−0.15	0.007	0.04	0.003	ND	-	−0.22	0.001
p-cymene	−4.31	0.003	−3.78	0.001	−1.47	0.027	−0.57	0.001	−1.92	<0.0001
hexyl acetate	−0.94	<0.0001	−0.04	0.398	−0.02	0.728	−0.05	0.346	−0.53	0.001
terpinolene	−0.06	0.714	−1.16	0.001	−0.01	0.976	−0.12	0.005	−0.37	0.0001
2-octanone	−0.09	0.390	−0.43	0.099	0.01	0.721	0.12	0.680	−0.11	0.271
octanal	0.003	0.970	0.40	0.392	−0.39	0.002	−0.02	0.895	−1.09	0.009
(Z)-3-hexen-1-ol acetate	−0.78	<0.0001	0.02	0.625	−0.03	0.317	−0.07	0.056	−0.28	0.0001
6-methyl 5-hepten-2-one	0.78	0.001	0.09	0.397	0.07	0.310	0.10	0.256	−0.75	0.001
1-hexanol	22.11	0.004	−25.08	0.001	1.43	0.127	−1.58	0.429	−23.28	0.002
(E)-3-hexen-1-ol	2.31	0.0001	−0.83	0.001	0.13	0.480	0.32	0.091	−0.52	0.050
(Z)-3-hexen-1-ol	−0.95	<0.0001	0.04	0.574	0.02	0.516	−0.03	0.273	−0.28	0.001
2-nonanone	−1.45	0.017	−2.58	0.001	−0.28	0.124	−0.36	0.005	−2.64	0.0001
nonanal	−0.01	0.984	1.15	0.191	−0.78	0.063	0.18	0.577	−1.91	0.075
furfural	−1.48	0.340	0.80	0.059	2.19	0.048	1.40	0.185	−0.60	0.643
tetramethylbenzene 1.2.3.4	ND	-	ND	-	ND	-	−0.16	0.102	1.14	<0.0001
2-ethyl-1-hexanol	−0.01	0.862	0.48	0.012	1.67	<0.0001	0.46	0.0001	−2.54	0.0001
decanal	−0.20	0.214	1.83	0.319	−0.18	0.230	−0.19	0.114	−0.58	0.029
benzaldehyde	−0.61	0.0001	1.36	0.0001	0.51	0.001	0.82	0.024	−0.08	0.869
linalool	0.11	0.201	−0.19	0.065	0.09	0.102	0.23	0.006	−0.29	0.008
hexadecane	0.05	0.001	ND	-	ND	-	ND	-	ND	-
4-terpineol	1.03	0.001	1.18	0.0001	0.58	0.0001	0.06	0.392	−0.17	0.004
2-octen-1-ol acetate	−0.45	0.073	−0.96	0.005	0.02	0.870	−0.01	0.845	−0.40	0.0001
1-hexadecene	−0.11	0.469	−0.74	0.208	−0.47	0.148	−0.56	0.288	0.04	0.468
acetophenone	−0.19	0.085	0.20	0.139	−0.20	0.126	−0.36	0.078	−0.64	0.189
unidentified hydrocarbon	0.50	0.234	−1.24	0.676	−2.62	0.159	−1.27	0.472	−0.15	0.136
heptadecane	ND	-	−0.20	0.607	−0.25	0.201	−0.13	0.500	ND	-
α-terpineol	0.13	0. 252	0.20	0.047	0.09	0.161	0.10	0.091	−0.45	0.0001
zingiberene	0.19	0.033	−0.22	0.112	−0.06	0.144	−0.47	0.0001	−0.78	0.0001
β-bisabolene	0.00	1.000	−0.25	0.033	−0.10	0.188	−0.15	0.044	−0.35	0.0001
naphthalene	0.29	0.095	−0.11	0.225	−0.12	0.241	0.03	0.899	1.33	<0.0001
β-sesquiphellandrene	0.08	0.005	−0.18	0.077	0.01	0.902	−0.18	0.035	−0.36	0.001
α-curcumene	0.04	0.450	−0.20	0.111	−0.10	0.087	−0.64	0.002	−0.93	<0.0001
3.5-dimethylbenzaldehyde	0.08	0.038	−0.04	0.603	0.34	0.015	2.27	0.0001	0.88	0.010
anethole	−0.24	0.001	−0.05	0.066	0.01	0.842	−0.003	0.820	−0.10	0.0001
hexanoic acid	0.16	0.024	−0.14	0.116	0.15	0.272	0.14	0.263	−0.47	0.108
phenol	−0.04	0.050	0.01	0.621	−0.02	0.566	−0.03	0.587	−0.11	0.153
p-cresol	−0.02	0.251	−0.02	0.374	0.00	1.0	−0.01	0.795	−0.03	0.270
m-cresol	−0.09	0.172	−0.03	0.647	−0.02	0.799	−0.05	0.541	−0.17	0.144

Δ* indicates variation between juices at t_0_ and t_28_ within the same preservation treatment (µg/L of 2-octanol). Delta was calculated as the difference between the average values (for the three replicates) at t_0_ and t_28_. ND: not detected.
